# Identifying Food Deserts and People with Low Food Access, and Disparities in Dietary Habits and Health in Korea

**DOI:** 10.3390/ijerph17217936

**Published:** 2020-10-29

**Authors:** Sanghyo Kim, Kyei-Im Lee, Seong-Yoon Heo, Seung-Chul Noh

**Affiliations:** 1 Korea Rural Economic Institute, Naju 58321, Korea; lkilki@krei.re.kr (K.-I.L.); heo0411@krei.re.kr (S.-Y.H.); 2 Seoul Institute, Seoul 06756, Korea; nsc0203@si.re.kr

**Keywords:** food consumption, access to food, disparity, health outcomes, food insecurity

## Abstract

This study aims to identify the regions and people with low food access (LFA) for Korea at the national level and to examine disparities in food consumption, dietary behavior, and health outcome for those regions and people. Based on the distance to the nearest grocery store from residence, the regions and people with LFA are identified through geographical information system (GIS) analysis. To examine disparities between the regions and people with LFA and without LFA, a consumer survey is conducted and data from National Health and Nutrition Examination Survey and Community Health Survey are analyzed. This study found that there exists a serious access to food issue in Korea, especially for the aged. Moreover, there also exist significant disparities between the regions and people with and without LFA in the distance and one-way travel time to the grocery store that is mainly visited, frequency of offline/online grocery shopping, availability of various foods, dietary habits such as eating regularly, eating nutritionally balanced foods, and eating sufficient fruit/vegetable/whole grains, the acquisition and utilization of food-related information, and health outcomes. This study suggests that, to resolve such a serious food access problem, assistance policies, such as mobile grocery stores and lunch-box delivery, need to be activated in countries similar to Korea since this problem could potentially deteriorate the national medical finances as well as the regional and individual disparities.

## 1. Introduction

Household food security is strongly associated with food supply and demand. For households to be food-secured, a variety of foods should be sufficiently supplied to the market at affordable prices, and the households must have full access to the supplied foods both economically and physically. According to the Food and Agriculture Organization of the United Nations (FAO-UN), “food security exists when all people, at all times, have physical and economic access to sufficient, safe and nutritious food that meets their dietary needs and food reference for an active and healthy life” [1]. The four key elements of food security in the traditional definition by FAO-UN, including availability, access, utilization, and stability, have been treated as a target to be achieved in the international community as well as in Korea. 

Although household income level has been identified as the most critical factor in determining household food security, policy makers and researchers have recently shown more interest in the physical environment surrounding food consumption as a significant external determinant of food security of households. As this external factor is uncontrollable by household themselves, it is increasingly attracting the attention of policy makers. Physical (or geographical) constraints that consumers face on food purchases reduce their feasible choice set, and thus could lower their potential (consumer) utility. The degree of which the consumers’ utility is lowered depends on how serious the physical constraints they face are. Since food consumption is closely related to the health of the people and the national economy, policy makers in each country should be fully aware of current situation on how serious the constraints are as well as of the related issues such as economic power to consume, accessibility to foods, food prices, availability of diverse foods, and related health outcomes.

The environment in which geographical constraints limit household food security is often called food deserts in the literature. Many researchers have investigated accessibility to grocery stores, inconvenience of grocery shopping, and inequalities and various differences between food deserts and non-food deserts. Morland et al. [2] and Rose and Richards [3] examined the number and density of the grocery stores that sell a variety of foods including healthy, functional foods as well as fresh and nutritious foods in each region. They also investigated the difficulty to visiting to those grocery stores. Rose and Richards [3], Chung and Myers [4], Hendrickson et al. [5], Powell et al. [6], and Zenk et al. [7] identified the factors that influence accessibility to grocery stores such as provision of local transportation, possibility of and potential risks in walking to grocery stores, restriction due to the business hours, single-parent household, and race and ethnicity. Powell et al. [6] and Zenk et al. [7] analyzed disparities and gaps in food deserts by examining the relationship between availability of grocery stores and racial/ethnic/socio-economic status. Tetzro et al. [8] also found that the distance, travel time, and transportation mode to grocery stores are the major factors influencing inconvenience of grocery shopping. They found that the average distance to the grocery store selling fresh foods in rural areas was 2.7 times farther than in urban areas. This implied that people living in rural areas suffer from unequal difficulties in securing sufficient, diverse types of foods.

Many researchers have analyzed dietary life and health outcome in food deserts or economically poor areas [4,5,9,10,11]. Cotterill and Franklin [12] and Weinberg [13] found that physical accessibility to grocery stores is correlated with prevalence of diabetes, heart diseases and cancer that are closely related to dietary habits in the regions where low-income households reside. Lewis et al. [14] concluded that the food environment in areas where low-income households reside hampers healthy dietary life. In addition, Lopez [15] investigated the relation between accessibility to grocery stores and the risk of obesity. Schafft et al. [16] scrutinized the relationship between living in food deserts and children’s body mass index. Thomsen et al. [17] examined the association of living in food deserts with body mass index of elementary school students. Fitzpatrick et al. [18] analyzed the relationship among living in food deserts, possession of own vehicles, and food insecurity. The existence of food deserts can result in significant regional inequalities in access to healthy foods [2,3,7], and accordingly cause serious health problems especially for the low-income households [12,13]. The regional inequality could also deteriorate societal integration and sustainability.

Due to its critical negative impacts, studies on food deserts have been conducted in many countries, including the United States, the United Kingdom, Japan, Canada, New Zealand, Australia, and Japan. For example, studies conducted in the United Kingdom include researches on the number of grocery stores in areas where low-income households reside [19,20], and studies on the availability of healthy foods [21,22]. Studies conducted in Canada include those about the number of grocery stores in areas by income level, the distance to grocery stores in rural and urban areas, and food prices by region [23,24,25,26,27,28,29].

Most of the studies introduced above have been conducted in a regional basis. National-level studies on food deserts have more recently been conducted. The U.S. Department of Agriculture’s Economic Research Service (USDA ERS) [30] conducted a study based on “the Food, Conservation, and Energy Act of 2008.” USDA ERS noted the significant impact of food deserts on prevalence of obesity and diet-related diseases in situation where obesity and diet-related diseases are emerging as a major public health threat. Based on this judgement, USDA ERS [30] conducted a national-level study on food deserts by using both individual-level survey and regional approach. In the regional approach, GIS analysis was implemented for the entire U.S. territory to divide it into unit grids of 1 km^2^, and the nationwide supermarket list data are used to measure the distance to the nearest supermarket from the center of each unit grid. Based on the measured distance, each area was classified into an area of high accessibility (the distance to the nearest supermarket not farther than 0.5 miles), an area of intermediate accessibility (0.5 to 1 mile), and an area of low accessibility (1 mile or farther). In Japan, as the issue of accessibility to foods has emerged in the background of the world’s highest proportion of the elderly population and the decreasing number of grocery stores, the Ministry of Agriculture, Forestry, and Fisheries (MAFF) has established and enforced an important policy research task of ‘difficulty in accessibility to foods’ since 2010 [31]. In 2012, the Policy Research Institute of MAFF published ‘Current Food Accessibility and Responding Task - in relation to Food Deserts’ and used GIS approach to build a food accessibility map for the entire territory of Japan.

The review of previous studies suggested that many regions in many countries have problems with access to foods and these can cause various negative impacts. For this reason, many countries have increasingly investigated the existence, severity, and related issues of food deserts. For Korea as well, it is highly urgent to examine the physical environment surrounding food consumption for two reasons. First, although the area of Seoul which is the capital city of Korea, and Gyeonggi-do surrounding Seoul accounts for only about 11.8% of the entire territory of Korea, more than half of the Korean population reside in the area (Statistics Korea) [32]. As more and more people gather and live in the capital area, the likelihood of occurrence of food deserts will increase and derivative problems will intensify. Secondly, Korea is making the fastest progress towards an aging society in the world. The proportion of elderly people is increasing rapidly from 7.2% in 2000 to 15.7% in 2020 and is expected to reach 33.9% in 2040 and 43.9% in 2060 (Statistics Korea). While the income level is the most important factor limiting household food security, the influence of demographic changes such as aging and the rapid increase in the proportion of single-person households is becoming greater nowadays. A critical problem here is that the number of the aged with mobility and ‘access to digital’ problems is also increasing in Korea. Combining these problems of the increasing elderly population with its associated physical constraints, grocery shopping becomes one of the most challenging tasks for the aged in Korea.

However, the physical environment surrounding food consumption has not been studied much in Korea. Only a few studies were conducted by the Rural Development Administration [33], namely those of Kim et al. [34], Chang et al. [35], and Jin and Kim [36]. The study in [33] conducted a pilot survey of the subjective perception of people and households on the environment surrounding food consumption by selecting 503 participants from 151 households in a single city, Hwaseong in Gyeonggi-do. They confirmed a significant disparity between rural and urban areas in food consumption behaviors that are associated with the food environment such as preferred transportation method, average travel time and distance to grocery store, the number of grocery stores that are visited, and ease of food purchase. They argued that this type of survey needs to be extended to the national level to provide policy insights. Kim et al. (2014) focused on the ‘dine-out environment.’ Using GIS approach for 275 people living in two urban areas and one rural area, the distribution and density of, and accessibility to restaurants were analyzed. They found that the density of restaurants was relatively higher in urban areas and the accessibility to non-Korean restaurants in urban areas and to Korean restaurants in rural areas were in a relatively acceptable level. Chang et al. (2014) developed a survey tool for measuring the ‘food accessibility’ and verified its reliability. The survey items suggested to be necessary include the general characteristics of grocery stores (whether or not they sell alcohol and tobacco, payment method, customer information, etc.), the geographical proximity to grocery store (availability, address, accessibility, parking), availability of various foods (eco-friendly products, healthy and functional foods, fresh vegetables and fruits, etc.).

Even though the term of food deserts is widely used in the literature, applying it for the case of Korea may not be appropriate not only because Korea’s territory is physically small but also because there are geographically no actual ‘desert areas’ in Korea. In this study, an environment with serious physical constraints that prevent people from achieving and pursuing food security is specified as ‘the region with low food access (LFA)’. Further, the people living in the region with LFA are referred to as ‘the people with LFA.’ There are a growing number of studies on LFA at individual and regional level in Korea, however, to the author’s knowledge, no national level study has been conducted yet. Identification of the regions and people with LFA is important as it will be the starting point of the designs of relevant policies to resolve the physical constraint issue addressed above. This study aims to identify the regions and people with LFA for Korea at the national level, and examine disparities in food consumption, dietary behavior and health outcome observed for those regions and people. It is expected that this study serves policy makers as the first national-level LFA study in Korea. For example, this study will provide basic information for domestic food assistance programs in Korea. Also, Korea is an appropriate country to conduct such a research as it is in transition to a more developed country with a recent large body of laws and policies aimed at the protection of consumers’ rights. Thus, the investigation on LFA for Korea is expected to have implications to countries in a similar cultural, sociodemographic, and economic status and/or transition.

## 2. Materials and Methods

### 2.1. Geographical Information System Anslysis

#### 2.1.1. Data and Scenario

**Figure 1:**
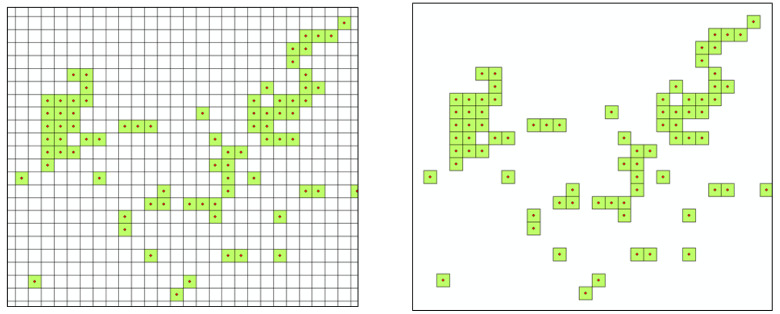
100 m × 100 m grid, entire set of grids (**left**), a subset of the grids with residents (**right**).

Based on the distance to the nearest grocery store, the regions and people with LFA are identified through geographical information system (GIS) analysis. To examine the accessibility associated with food consumption, information on the population living in geographical units smaller than the smallest (or lowest level) administrative district (Eup, Myeon, or Dong in Korean) is analyzed using the National Geographic Information Institute data. The National Geographic Information Platform operated by the National Geographic Information Institute divides the entire territory of Korea into a set of 100 m × 100 m grids and provides information of the population living in each grid by gender and age. According to this data, South Korea is geographically divided into approximately 41 million 100 m × 100 m grids among which only 986,000 grids have residents (see Figure 1). As of the end of June 2020, the total population of Korea was 51,839,852, and thus approximately 53 people lived in each grid of the 986,000 grids with residents.

On the other hand, information about the size and type as well as latitude and longitude of grocery stores is also necessary for examining, based on the distance between residential area and the nearest grocery store, the accessibility to foods. Information on grocery stores provided by the business database of the Small Enterprise and Market Service is used. The business database is classified into 20 categories, 238 subcategories, and 3286 sub-subcategories of businesses, and contains information on about 3 million businesses among which 76,207 are grocery stores.

The accessibility to foods depends on the type and size of grocery stores that are considered. For an extreme example, if we only consider warehouse clubs such as Costco and Sam’s Club in the United States as grocery store, then there will be a huge number of people with LFA. For this reason, in this study the distance to the nearest grocery store is measured for three cases (or scenarios) with different types of grocery stores. For Case 1, only 35,885 grocery stores (e.g., hypermarkets, department stores, etc.) that sell a variety of foods including fresh and functional foods out of total 76,207 grocery stores included in the business database of the Small Enterprise and Market Service are defined as grocery store. Case 1 can be regarded as the most conservative (or most pessimistic) example of evaluating the accessibility to foods. The second case has 48,495 grocery stores that do not only include the grocery stores under Case 1, but also include small and local grocery stores. Small and local grocery stores are, in general, smaller than the grocery stores under Case 1. These grocery stores are run by individual owners and are generally known to sell essential food items. Finally, Case 3 includes all the grocery stores including convenience stores as well as the grocery stores under Cases 1 and 2 (see Table 1). Case 3, contrary to Case 1, can be regarded as the most optimistic example of evaluating the accessibility to foods.

#### 2.1.2. Identification of the Regions and People with LFA

In this study, the regions and people with LFA are identified based on the distance from their residence to the nearest grocery store. To measure the distance, three pieces of information were needed. The first information required is the location of residence, and the second is the information of the location of the entire set of grocery stores. The third piece of information required is how far the distance is to be the determinative reference for evaluating the accessibility to foods. Because the address and demographics of each household are not public information, it was not possible to examine the accessibility to foods at the household level. This study thus used data provided by the National Geographic Information Platform on the number of people living in each of the 100 m × 100 m grids by age and gender. One inevitable assumption was that all people living in one of the 100 m × 100 m grids reside in the center of the grid. Considering that the 100 m × 100 m grids dividing the entire territory of Korea into 41 million grids are very tiny geographical units, this assumption is acceptable. Accordingly, the location of residence of people living in grid *g* is expressed in Equation (1):

*LOCg* = (*lat*(*c*(*g*)), *long*(*c*(*g*))) (1)

where *LOCg* is the location of grid *g*, *lat*(*c*(*g*)) is the latitude of the center of grid *g*, and *long*(*c*(*g*)) is the longitude of the center of grid *g*.

On the other hand, the latitude and longitude information included in the business database is provided by the Small Enterprise and Market Service to examine the location of grocery stores. The location of grocery store *j* is expressed in Equation (2):

*LOCj* = (*lat*(*j*), *long*(*j*)) (2)

where *LOCj* is the location of grocery store *j*, *lat*(*j*) is the latitude of grocery store *j*, and *long*(*j*) is the longitude of grocery store *j*.

**Figure 2:**
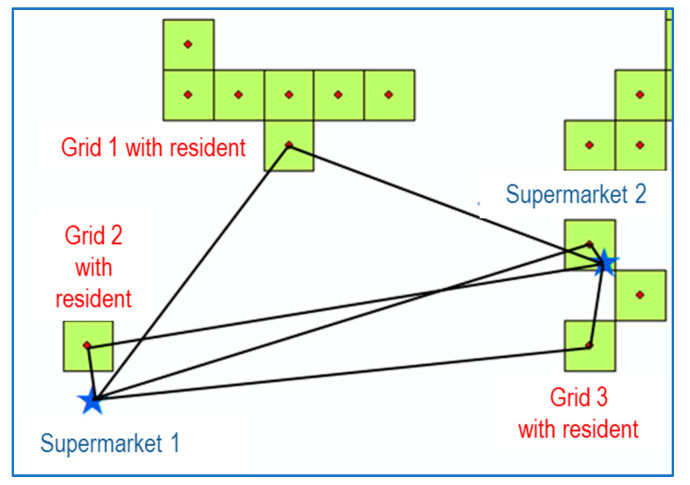
Measurement of the distance from the center of grid with residents to the nearest grocery store (supermarket).

The distance from a residence to a grocery store is calculated based on the straight-line distance between the two points. It would be more appropriate to apply actual environment related to roads, for example, the road network, average speed of each road, and traffic signal system in measuring (true) accessibility between a residence and a grocery store. However, the straight-line distance measurement between the two points of interest is inevitably adopted because no such data were available. After measuring the straight-line distance between a residence (here, the center of grid *g*) and each of all the grocery stores *j* = 1, 2, 3, …, *J* for each grid *g*, the minimum value was specified as the distance to the nearest grocery store for that grid (see Equation (3) and Figure 2). In this procedure, the ‘Near’ function among the functions of the spatial information analysis tool ‘ArcMap’ was used.

(3) $$NEAR\{g,\;J\}=\underset{j\in \left\{1,\;2,\;3,\;\ldots ,\;J\right\}}{\min}\sqrt{({\left|lat\left(j\right)-lat\left(c\left(g\right)\right)\right|}^{2}}+{\left|long\left(j\right)-long\left(c\left(g\right)\right)\right|}^{2})$$

**Figure 3:**
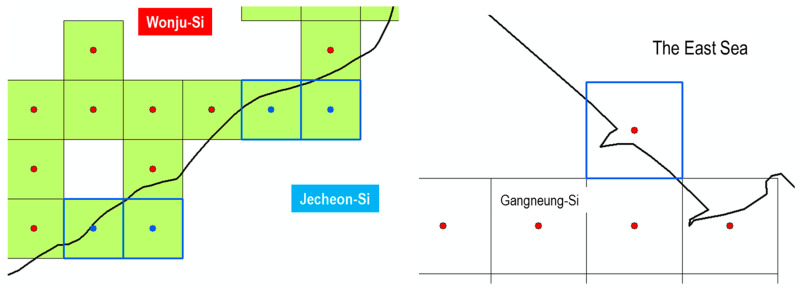
Assignment of 986,000 grids to administrative districts (Si, Gun, or Gu).

South Korea is divided into 229 administrative districts, known as lower-level districts (Si, Gun, and Gu in Korean), and each lower-level administrative district is composed of 4306 grids on average. As potentially useful policy information is the proportion of the people with LFA by lower-level administrative district, not for the tiny grid level, each grid needs to be assigned into an administrative district. Two rules are applied in this assignment task. First, a grid is assigned to the lower-level administrative district to which the center of the grid belongs. The grids are square in shape and do not exactly match the boundary of administrative districts. Therefore, this rule means that one grid is assigned to a certain administrative district depending on to which administrative district a larger portion of the grid belongs. Second, if the center of the grid is out of the land, then the grid is assigned into the administrative district that the grid touches (see Figure 3). The ‘intersect’ function among the functions of spatial information analysis tool ‘ArcMap’ was used for this procedure.

Once the distance from the center of each grid to the nearest grocery store is measured and thus known, the next step is to determine whether the people living in each grid suffer from LFA. This decision depends on the answer to the following question: how far from the nearest grocery store can food access be considered low? Although there is no specified range about the sphere of on-foot living, the concept of ‘neighborhood unit’ has generally been used in urban planning, which was created by the American C. A. Perry in the 1920s. Neighborhood unit is a residential unit allowing residents to move to the center of the unit on foot and to share public services including elementary schools, shops, parks and green areas. The size of a neighborhood unit in Korea is generally based on the distance which children can travel to their elementary school on foot and is therefore within a 400 to 800 m radius. Lee and Park [37] defined the radius of neighborhood unit for Korea as 338.6 to 506.4 m based on the distance to parks, bus stops, markets, grocery stores and halls for the aged in their study about aged-friendly residential area. By assuming that grocery stores should be within walking distance and by referring to the suggestions from previous studies, this study identifies the grids of which the distance to the nearest grocery store is farther than 500 m as the region with LFA, and the population living in such a grid or region are defined as the people with LFA.

### 2.2. Survey for Accessbility to Foods, Food Consumption Behavior, and Dietary Habits

To evaluate the differences in food consumption and dietary habits between the people with and without LFA, this study conducted a survey for 1100 consumers. This survey was implemented through face-to-face interviews for adults aged 20 s through 60 s across the country by using a structured questionnaire. For a comparison purpose, 100 people among 1100 respondents were sampled from the regions with LFA. This selection of the people with LFA was based on the result of GIS analysis described above. More specifically, 20 respondents sampled from Ongjin-gun, Sinan-gun, Hampyeong-gun, Jinan-gun, and Gunwi-gun, respectively, participated in this survey.

For a comparison purpose, the people without LFA (n = 1000) are classified into three sub-groups: without-LFA rural residents (n = 100), mid-sized urban residents (n = 440), and metropolitan residents (n = 460). Attributes of food consumption and dietary habits examined in this study include the frequency of purchasing entire foods, fruits, vegetables, and meats, the rate of skipping breakfast, the rate of eating regular meals, the rate of eating meals alone, transportation mode mainly used for food shopping, distance and one-way travel time to the grocery store that is mainly visited, availability of a variety of foods, and the subjective evaluation on shopping inconvenience and on current dietary habits.

As seen in Table 2, females account for 49.5% of 1100 survey respondents, and respondents with university or higher education level account for 47.7%. The respondents with monthly household income not more than 3 million Won account for about 34%, and the respondents with monthly household income 3–5 million Won and more than 5 million Won account for 48% and 18%, respectively. Residents living in ‘Dong’ areas (usually urban areas) account for 72.7%, and those in ‘Eub/Myeon’ areas (usually rural areas) account for 27.3%, implying a balanced sample.

In addition to the survey implemented for this study, the Consumer Behavior Survey for Foods (CBSF) is also analyzed to test disparities in dietary behaviors [38]. CBSF has been annually conducted by the Korea Rural Economic Institute since 2013, and is known to be a representative national survey on food consumption in Korea.

### 2.3. Analysis of Regional and National Health Survey

The survey introduced in the previous section was conducted for a relatively small sample of consumers, and hence did not completely represent all lower-level administrative districts as well as the entire Korean population. To compensate for this representativeness limitation, data from the Community Health Survey (CHS) [39] were analyzed to examine if there exist disparities in health outcomes between the regions with and without LFA. The CHS has been annually conducted by the Korea Centers for Disease Control and Prevention in collaboration with 255 public health centers under lower-level administrative districts across the country since 2008. The sample size for each lower-level district is sufficient (about 900) and thus strengthens the representativeness. Because the accessibility to foods can be measured for each lower-level administrative district based on the proportion of the regions (or grids) of the lower-level district in this study, it is possible to identify the lower-level administrative districts with the lowest food access. Since the CHS provides representative statistics of each lower-level district, useful comparisons of the lower-level districts with LFA with national average can also be made. This study utilized the questions related to dietary habits and health outcome such as reading nutrition labels, the quality of life index, obesity (actual measurement), and diagnoses of hypertension and diabetes to examine the dietary habits and health outcomes in the regions with LFA by comparing the averages in top 10 regions with the lowest food access identified in the GIS analysis with the national averages calculated using the Korean Health and Nutrition Examination Survey (KNHANES) data [40].

## 3. Results

### 3.1. Accessibility to Foods in Korea

#### 3.1.1. Average Distance to the Nearest Grocery Store by Case

For Case 1, which is the most conservative case, the average distance to the nearest grocery store was about 1.8 km. Since Case 1 is the most pessimistic scenario in terms of the accessibility to foods, this implies that the distance to the nearest grocery store that sells almost all the foods consumers want at reasonable prices is about 1.8 km on average for the Korean households. One-way 1.8 km is a distance for which approximately 30 min are taken on foot. If consumers do not have their own vehicle and/or if public transportation is not available for grocery shopping, the distance of 1.8 km requires consumers to spend about one hour for round trip. Moreover, consumers should carry heavy grocery bags when they return. It is not a short distance. Looking at Case 3 results, the most optimistic scenario, the average distance to the nearest grocery store is 1.46 km, not a huge difference. And, the average distance significantly varies by the lower-level administrative district, implying a potential existence of regional disparities both in food access and consequently in food consumption and dietary life. The correlation coefficient between the population density (based on the Population and Housing Census in 2015) and the average distance to the nearest grocery store was −0.69. As higher population densities result in more grocery stores available, more grocery stores may result in shorter distances to the nearest grocery store. Therefore, areas with lower population density may suffer from a more serious food access problem. This analysis also found that, while the average distance to the nearest grocery store in Seoul is as short as 199 m, it is quite long in Gyeongsangbuk-do, i.e., 2555 m, which is the farthest (based on Case 1) (see Table 3).

#### 3.1.2. The Number of People with LFA and its Proportion

All people living in the grid where the distance to the nearest grocery store is 500 m or farther were considered as the people with LFA in this study. Because each grid was assigned into a lower-level administrative district, it was possible to calculate the number of the people with LFA by upper-level administrative district (metropolitan Si and Do in Korean) and its proportion among the entire population of each upper-level district. Based on Case 1, the number of the people with LFA is about 6.3 million in Korea, accounting for about 12.3% of the total population of Korea. This number is even greater than the national statistics for the number of households that are food insecure, implying a potential underestimation of the social problem associated with the accessibility to foods. A more serious problem is the population aged 65 or more (Table 4). More than 20% of this age group turn out to face LFA constraint even in the most optimistic case.

**Figure 4:**
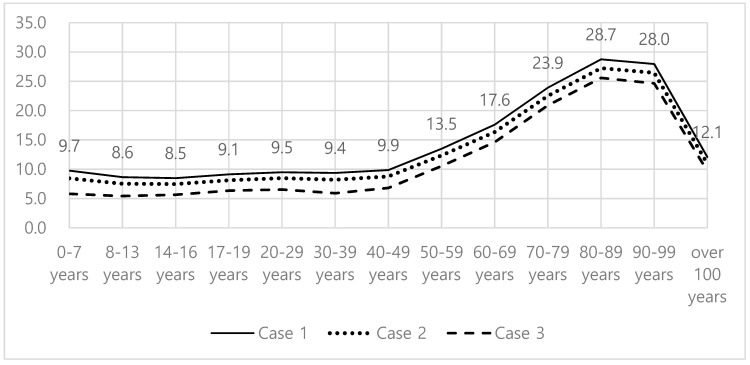
Proportion of the people with LFA by age group (unit: %).

The number of people aged 65 or higher living in the regions with LFA is approximately 1.56 million, accounting for 23.2% of the entire number of people aged 65 or higher in Korea (Table 4). The analysis of the proportion of the people with LFA in each age group indicates that the proportion of the people with LFA is about 10% for the age groups younger than 40, but begins to increase for the age groups older than 40, exceeding 20% for the people aged 80 or higher (Figure 4). This finding suggests that the LFA issue in Korea is in line with an aging issue.

**Figure 5:**
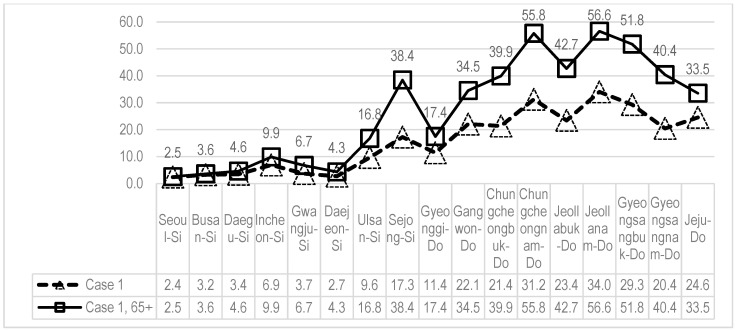
Proportion of the people with LFA by upper-level administrative district (unit: %).

The proportion of the people with LFA significantly varies by upper-level administrative district (metropolitan Si and Do). For metropolitan Si areas, the proportion is not higher than 10% and is even smaller than 4% except for Incheon and Ulsan. On the other hand, the proportion is higher than 20% for most Do areas including Jeollanam-do and Gyeongsangbuk-do. The proportion of the people with LFA aged 65 years or higher is higher than 50% for several Do areas including Jeollanam-do, Chungcheongnam-do, and Gyeongsangbuk-do (based on Case 1) (see Figure 5).

#### 3.1.3. Map for Accessibility to Foods and Identification of the Regions with the Lowes Food Access

As remarked in the earlier section, each 100 m × 100 m grid is assigned to lower-level administrative district. Korea is composed of 229 lower-level districts (Si, Gun and Gu), and a lower-level district is composed of approximately 4306 100 m × 100 m grids on average. In a grid, about 53 people live on average. A grid (or a region) is specified as the grid with LFA when the distance from its center to the nearest grocery store is 500 m or farther. And, all people living in the grid with LFA are counted as people with LFA. Accessibility to foods of a lower-level administrative district is measured based on the ratio of the people with LFA to the entire population living in the lower-level district. That is, the accessibility to foods of a lower-level district is a continuous metric ranging from zero to one. This method is applied to calculating the accessibility to foods of each lower-level districts (Si, Gun, and Gu) as well as upper-level administrative districts (metropolitan Si and Do).

Table 5 illustrates top 20 lower-level administrative districts with the lowest food access. These include Ongjin, Sinan, Gunwi, Seongju, Hampyeong, Sancheong, Goesan, Jinan, Uiryeong, Ganghwa, Jindo, Hadong, Imsil, Cheongdo, Haman, Bonghwa, Jangsu, Uiseong, Hapcheon, and Yeongyang. Among these 20 lower-level administrative districts, Gunwi, Seongju, Sancheong, Uiryeong, Ganghwa, Ongjin, Sinan, Hampyeong, Jinan, and Goesan were selected as 10 lower-level administrative districts with LFA for comparison of their average dietary habits and health outcomes with the average of Korea. For these top 20 lower-level administrative districts with LFA, this study found that more than 60% of entire population was living in the regions with LFA.

In Sinan, Gunwi and Hampyeong, the proportion of the people with LFA was higher than 70%, implying a serious physical constraint issue. Ongjin which ranked the highest in Case 1 was on the place slightly lower in Cases 2 and 3. It is because there were other types of grocery stores and convenience stores of Cases 2 and 3 available in Ongjin although there were not many grocery stores of Case 1 type. In consideration that 12.3% of the total population of Korea faces the LFA constraint, the proportion suggested in Table 5 is certainly high. Because it is likely that food consumers living in these regions with LFA suffer from difficulties in purchasing sufficient, various, and fresh foods at reasonable prices, policy makers should be interested in this finding.

**Figure 6:**
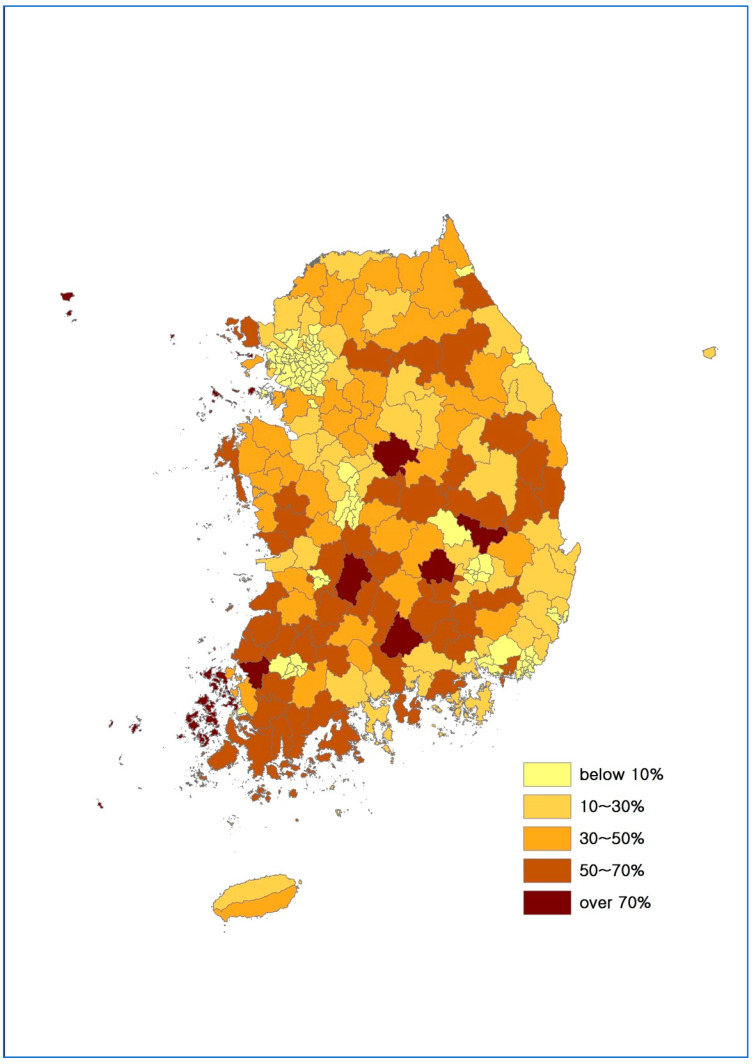
Map for Accessibility to Foods of Korea, Case 1.

Figure 6 illustrates the map for the accessibility to foods of Korea. This is the first national-level map depicting the accessibility constraint of the entire Korea. This map is drawn for the 229 lower-level administrative districts based on the proportion of populations living in the regions with LFA (grids). A direct implication is that the southwestern regions and islands of Korea suffer from more serious LFA issue, while this issue is less serious in metropolitan areas including Seoul and Busan.

### 3.2. Disparities in Dietary Habits and Health by Accessibility to Foods

#### 3.2.1. Disparities in Dietary Habits and Food Consumption Behaviors

One of the purposes of this study is to investigate the differences in food consumption between the people living in the regions with and without LFA. The GIS analysis explicitly identified the regions with LFA based on the distance to the nearest grocery store, and the questionnaire survey focused on dietary habits and food consumption behaviors as well as consumers’ subjective perception on the distance and travel time to grocery stores. According to the survey, the one-way travel time to the grocery store mainly visited by the people with LFA was 14.8 min on average. This is at least 40% longer than the one-way travel time for the people living in without-LFA rural areas (11.4 min), mid-sized urban areas (11.2 min), and metropolitan areas (10.4 min), respectively (see Table 6). This analysis also found that the distance to the grocery store mainly visited was higher for the average of top 5 lower-level administrative districts with LFA (7.2 km) than without-LFA rural areas (3.5 km), mid-sized urban areas (3.6 km), and metropolitan areas (2.0 km), respectively (see Table 6). This disparity in distance and one-way travel time to go to the grocery store that is mainly used for grocery shopping between the regions with and without LFA was statistically significant at the one percent level.

Since the distance to the nearest grocery store is farther for the people with LFA, the direct costs required for grocery shopping such as time and transportation costs will likely increase. A person who has physical difficulties in walking will also have to bear additional costs derived from inconveniences of moving and carrying things above the time and transportation costs. Because foods, especially agricultural products are perishable over time, there exists, for each consumer, an (unobservable) proper shopping cycle or appropriate amount of foods purchased per shopping. It is highly unlikely that the people with LFA, due to their environmental limitations, purchase the appropriate amount of foods within their own proper shopping cycle. Such limitations in grocery shopping can be examined by looking at the difference in grocery shopping cycles, presented in Table 7. In the top five lower-level administrative districts with LFA, approximately 70% of people tend to go grocery shopping at least once per week, while it is higher than 80% in the rest of regions. In contrast, 91.6% of people living in (without-LFA) metropolitan areas go grocery shopping at least once per week. This disparity in the frequency of grocery shopping is statistically significant at one percent level. A longer cycle of grocery shopping may involve two possible situations. First, a longer shopping cycle means that consumers purchase foods more than needed, probably more than the proper amount, at one time. In this situation, their utility is likely to be reduced and the possibility of suffering from issues related to freshness, quality and safety is likely to increase. Secondly, it is also likely that their diets are insufficient without ensuring enough foods required for everyday life. If ether is the case, we could conclude that the LFA issue is associated with quantitative lack of food consumption either in a short term or mid to long term.

Even though accessibility to foods is lower for offline grocery shopping (i.e., actually going to grocery store for food shopping), people might be able to purchase foods using online websites (i.e., online orders), implying ‘offline LFA’ may not be a problem for some people (of course, food deliveries from online shopping malls still involve issues of freshness or safety of foods). However, according to the survey, approximately 73% among people living in the regions with LFA never purchase foods via online, indicating a difference of more than 10%p in comparison with 60.5% and 60.7%, respectively, in mid-sized urban areas and metropolitan areas (see Table 8). This disparity in the frequency of online grocery shopping is also statistically significant at the one percent level. If people living in the regions with LFA are more vulnerable in terms of the offline LFA than any other regions (mid-sized urban area, metropolitan area), online shopping could be an alternative way for grocery shopping. However, the survey results show that online grocery shopping currently never seems an effective way to improve grocery shopping for the regions with LFA. In other words, it seems that the offline LFA problem might be positively correlated with the ‘online LFA’.

The LFA constraint means that people cannot purchase sufficient fresh and various foods at reasonable prices whenever people need them. Although a person wants to take dietary supplement or health functional foods, people living in the regions with LFA may have a difficulty in finding the grocery store that sells those foods. In addition to the case where it is impossible to purchase certain types of foods at all (impossibility issue), there is also a limitation of low diversity for certain food items that can be purchased, fruits for example (diversity issue). All these constraints will interfere with balanced nutrition intake. While the issue for frequency of online/offline grocery shopping is associated with the quantitative problem of food consumption that the people with LFA face, the impossibility and diversity issue is related to the qualitative problem of food consumption. Table 9 represents the subjective evaluation on the availability of a variety of foods needed for nutritionally balanced dietary life in the grocery store that is mainly used for grocery shopping. Approximately 88% of the people with LFA rated their accessibility to foods as fully available in terms of the diversity of foods, while it is 97% for people living in the regions without LFA. This disparity in the availability of a variety of foods is also statistically significant at the one percent level.

**Figure 7:**
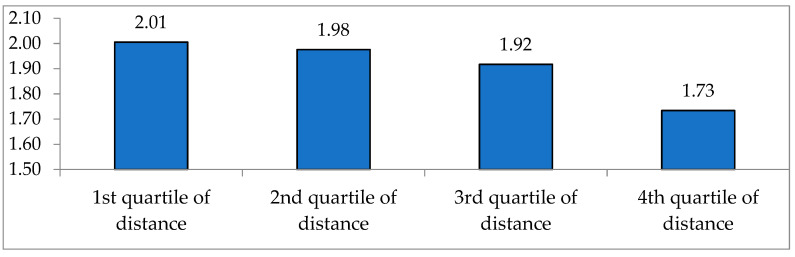
Diversity of fruits consumed during the past week by quartile of distance to the nearest grocery store. Note: *p*-value = 0.000 in t-test Source: Consumer Behavior Survey for Foods [38].

The qualitative aspect of LFA can be found out indirectly by examining how many items of fruit are purchased and eaten during the past one week. Figure 7 depicts the number of items of fruit that are eaten during the past one week by quartile of distance to the nearest grocery store. The distance to the nearest grocery store is obtained from the stated responses of the questionnaire survey. While the average number is 2.01 for the consumers whose distance to the nearest grocery store is the closest (1st quartile), it is dramatically reduced to 1.73 for the consumers of 4th quartile distance. Even though this is also significant evidence that supports the relationship between the LFA constraint and the diversity of diets, careful attention is required in interpreting these numbers as the distance to the nearest grocery store might be correlated with the level of income and/or age.

**Figure 8:**
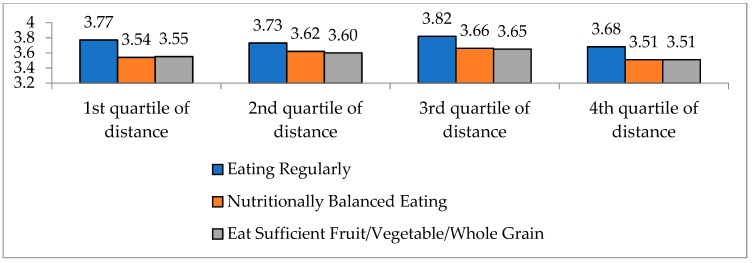
Eating habits by quartile of distance to the nearest grocery store. Note: *p*-value = 0.000 in t-test Source: Consumer Behavior Survey for Foods [38].

The LFA constraint affects dietary habits such as eating meals regularly, eating nutritionally balanced diets, and eating sufficient fruit/vegetable/whole grain. For example, if it is difficult to purchase needed foods at a proper time or if it is hard to purchase all the necessary food items at one time for some people, then their dietary lives could be involuntarily irregular or unintentionally imbalanced in terms of nutrition intake. Figure 8 indicates the subjective evaluation on dietary life using a five-point Likert scale. Overall, the subjective assessment of dietary life is slightly lower for the people with the lowest food access (4th quartile) than the other groups (1st~3rd quartile groups). For example, the evaluation on regular eating is 3.77 and 3.68 for 1st quartile and 4th quartile distance group, respectively. This disparity is statistically significant at the one percent level.

**Figure 9:**
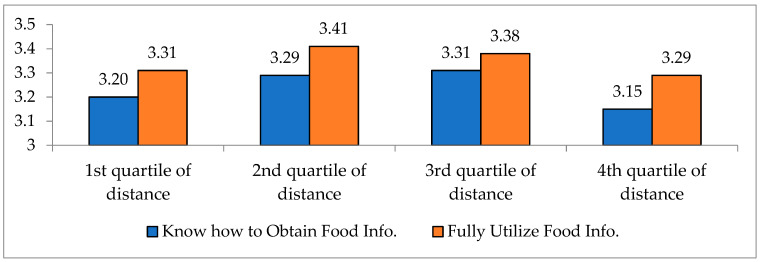
Acquisition and utilization of food-related information by quartile of distance to the nearest grocery store. Note: *p*-value = 0.000 in t-test Source: Consumer Behavior Survey for Foods [38].

It turns out that the LFA constraint is associated with the acquisition and utilization of the information related to foods and dietary life. The 5-level Likert scale questions are asked to measure how people acquire food-related information, and whether they fully utilize the acquired information. Figure 9 represents the results. Overall, the acquisition and utilization of food-related information is better for the people with the lowest food access (4th quartile) than the other groups (1st~3rd quartile groups). For the consumers living farthest (4th quartile) from their main grocery stores, the evaluation on whether they know how to obtain the food-related information is 3.15 out of 5.00, quite low compared to other consumer groups. Also, the information utilization is 3.29 out of 5.00 for the people with the lowest food access (4th quartile), while it is 3.37 on average for the other groups (1st~3rd quartile groups). This disparity is statistically significant at one percent level. These findings could imply that the LFA constraint is related to acquiring and utilizing food-related information, thus it will be more appropriate to focus on the groups of people with LFA when providing the food-related information and dietary education.

#### 3.2.2. Disparities in Health and Well-Being

As remarked above, the people facing LFA constraint cannot purchase foods within a proper purchase cycle, and thus are highly unlikely to purchase a proper amount of foods. This is the quantitative impact of the LFA constraint. This study also found that the LFA constraint could have influence on the quality of food consumption and dietary habits. These quantitative and qualitative impacts of the LFA constraint on food consumption and dietary habits can consequently have influence on people’s health, of course. To evaluate its impact on health, this study analyzed several health and quality of life indicators. Health indicators include the prevalence of high blood pressure, diabetes, and obesity, as they are all diet-related. Meanwhile, because health issues are causally linked to the quality of life, the EQ-5D index designed to measure the quality of life is also examined. Although the impact of the LFA constraint on health or quality of life should be examined in the long run, it is examined indirectly in this cross-sectional analysis based on the assumption that there has been no dramatic change in food consumption environment (or LFA constraint) across time.

Table 10 illustrates the prevalence of high blood pressure, diabetes, and obesity, and EQ-5D index based on KNHANES in 2017 for national average and the CHS for the regions with LFA. While approximately 26.9% of all Koreans suffer from high blood pressure as of 2017, quite higher proportion, 34.3%, of people living in top 10 lower-level administrative districts with LFA suffer from high blood pressure, which is 7.4%p higher. The prevalence of diabetes is 10.4% for all Koreans, but it is 12.9% for the people living in top 10 lower-level administrative districts with LFA. Moreover, the prevalence of obesity is 34.1% and 35.0%, respectively. The index for the quality of life, EQ-5D, is 0.963 for all Koreans but is much lower as 0.923 for the people living in top 10 lower-level administrative districts with LFA, showing a significant difference. Because people living in the regions with LFA highly unlikely maintain desirable food consumption in terms of the quantity and quality of foods they eat, unbalanced nutrient intake could be very likely, resulting consequently in the prevalence of diseases related to dietary life. Since this can sequentially have an influence on the overall quality of life of people, nationwide interest in related food policies is required for the regions and people with LFA in Korea.

Because the disparities in the prevalence of diet-related diseases and the quality of life indicator can result from heterogeneous population composition (e.g., high proportion of the elderly), an additional analysis has been made by age group. As can be seen in Table 11, the proportion of people reading the nutrition fact labels is 33.2% for all Koreans but is quite lower as 14.3% for the 10 lower-level administrative districts with LFA. Moreover, this finding holds for all age groups. In addition, this study also found that the quality of life varies by age as well as the LFA constraint. In most age groups, the quality of life index is similar, but for the age group of 70 or older it is quite lower for the top 10 regions than the national average of Koreans. The disparity in the prevalence of obesity seems to be an even greater problem for the age group of 40 or younger than the aged group. While 33.4% of people in their 30 s are obese, for all Koreans, it is 38.0% in the top 10 regions (see Table 11).

## 4. Conclusions

Household food security is strongly associated with food supply and demand. For households to have food security, a variety of foods should be sufficiently supplied to the market at affordable prices, and the households must have full access to the supplied foods both economically and physically. Although household income level has been identified as the most critical factor in determining household food security in the literature, policy makers and researchers have recently shown more interest in the accessibility to foods as a significant external determinant of food security of households.

It is highly urgent to examine the accessibility to foods for Korea, as grocery shopping becomes one of the most challenging tasks both for rural areas and for the elderly due to its fastest aging, mobility, and access to digital issue of the elderly, and centralization to capita area. However, the accessibility to foods arena has not been studied much in Korea. To the authors’ knowledge, no national level study has been conducted. Identification of the regions and people with LFA is particularly important as it will be the starting point of the designs of relevant food policies. This study aims to identify the regions and people with LFA for Korea at the national level, and examine disparities in food consumption, dietary behavior and health outcome observed for the regions and people with and without LFA. Korea is an appropriate country for such a case study as Korea is in transition to a more developed country with a recent large body of laws and policies aimed at the protection of consumers’ rights and right to foods. Hence, the investigation on the LFA constraint for Korea is expected to have implications to countries in a similar cultural, sociodemographic, and economic status and/or transition.

Based on the distance to the nearest grocery store, the regions and people with the LFA constraint are identified through GIS analysis. And a continuous metric for the accessibility to foods ranging from zero to one is granted for each lower-level and upper-level administrative district. Data from survey for consumers and from KNHANES and CHS are analyzed to investigate the disparities in food consumption behavior, dietary habits, and health outcome between the top five (or 10) lower-level administrative districts with LFA and the other regions.

It is shown that 6.3 million people accounting for 12.3% of all population of Korea live in the regions with LFA. A more serious problem is the population aged 65 or more. Higher than 20% of this age group turns out to suffer from the LFA constraint even in the most optimistic case. This finding suggests that the LFA constraint issue in Korea is quite an aging issue.

This study also found that the proportion of the people with LFA significantly varies by upper-level administrative district (metropolitan Si and Do). For metropolitan Si areas, the proportion is not higher than 10% and is even smaller than 4% except for Incheon and Ulsan. On the other hand, the proportion is higher than 20% for most Do areas.

Using the continuous metric for the accessibility to foods ranging from zero to one, this study has drawn the first national-level map depicting the accessibility to foods of the entire territory of Korea. This map is drawn for the 229 lower-level administrative districts based on the proportion of populations living in the regions (or grids) with LFA. This study found that the southwestern regions of Korea suffer from more serious LFA constraint. It turns out that there exist significant disparities between the regions with and without LFA in: (1) distance and one-way travel time to the grocery store that is mainly visited, (2) frequency of offline/online grocery shopping, (3) availability of a variety of foods, (4) the number of items of fruit that are eaten during the past one week, and (5) dietary habits, such as eating regularly, eating nutritionally balanced, eating sufficient fruit/vegetable/whole grain, and the acquisition and utilization of food-related information. Furthermore, this analysis found evidences supporting that the quality of life is lower and the prevalence of diet-related diseases is higher for the people living in the regions with LFA.

The findings from this study indicate that there exists a more serious LFA constraint problem in Korea than expected. Hence, policy makers’ attention is required for this environment issue as this could potentially be associated with regional and individual disparities in food consumption, nutrition intake, and health outcomes. Moreover, to achieve both a healthy life and food security, improving the food consumption environment as well as pursuing economic growth are required. The Korean government must address the urgent issues of population declination, fastest aging, and extinction of villages. To this end, the disparities in the accessibility to foods should be resolved, and the policies targeting the socially weak including the aged and the disabled should be more actively developed.

Japan that has focused on the food desert issue earlier than Korea has also developed various policies to resolve this environmental issue and thus is worth a reference. Specifically, exemplary strategies include opening small grocery stores in the regions with LFA, encouraging convenience stores to sell fresh foods, providing transportation mode such as community buses or mobile grocery shopping services, and providing more services of delivering lunch boxes for the people living in those regions.

This study has several limitations. First, this study measures the “straight-line” distance from the center of grid to the nearest grocery store. In order to appropriately reflect the actual accessibility between residence and grocery store, the road network map, signaling system and the average speed of roads should be considered, but the straight-line distance is simply used due to the unavailability of relevant data. Second, a strong assumption is applied: the entire population residing in a grid live at the center of the grid. Since the individual-level address data were not available, this assumption was inevitably made. However, because it is an extraordinarily strong assumption, relaxation of this assumption is needed in future research by obtaining individual-level data. Third, the influence of self-sufficiency or possibility of online order on food consumption in rural areas was not considered. In rural areas, many people involve production of foods or order foods using internet websites (online shopping). In this case, the LFA constraint may be not that serious for those people. Fourth, other factors that affect disparities in dietary habits and health outcome were not fully considered in this study. Average income or age might vary by the quartile of the distance to the nearest grocery store, for example, resulting in the possibility that the disparities stem not only from the LFA constraint but also from the socioeconomic status such as income and age. Further researches need to control those factors including disability, ability to walk, physical condition, and even access to vehicle and public transportation for food shopping. Fifth, this study investigated only a limited set of outcome variables such as the prevalence of diseases and acquisition and utilization of food information, and dietary habits like regular meals. The outcome variables need to be better organized and the related questions need to be better designed in the future study. Lastly, the disparities in health outcome were examined using cross sectional data, not the time series data. As health outcomes is kind of long-term impact, it is more appropriate to use time series data. Future studies are expected to address and improve these limitations.

Despite various limitations, this study contributed to grasping the current situation of Korea’s accessibility to foods, an important environmental topic, by drawing a map for the accessibility to foods at the national level for the first time. In addition, this study presents a contribution in that it empirically supported the fact that accessibility to foods could be related to food consumption and dietary behaviors, and consequently health outcomes.

## Figures and Tables

**Table 1:** Cases (Scenarios) according to the inclusion criteria of grocery stores.

Case	Number of Grocery Stores	Type of Grocery Stores Included
Case 1 (most pessimistic)	35,885	Department stores, supercenters, large supermarkets, marketplace/shopping malls, special stores for organic foods, discount food chains, farmer’s market
Case 2	48,495	Case 1 + small grocery stores, arcade/shopping centers, and local grocery stores
Case 3 (most optimistic)	76,207	Case 2 + convenience stores

**Table 2:** Demographics of survey respondents.

		Number of Observations	Ratio (%)
**Total**		1100	100.0
Gender	Male	555	50.5
	Female	545	49.5
Age	20∼29	178	16.2
	30∼39	211	19.2
	40∼49	250	22.7
	50∼59	247	22.5
	60∼74	214	19.4
Education	Less than high school	151	13.7
	High school	424	38.5
	University degree	516	46.9
	Graduate school degree	9	0.8
Monthly household income	Smaller than 1 million KRW	44	4.0
	1∼2 million KRW	115	10.5
	2∼3 million KRW	211	19.2
	3∼4 million KRW	269	24.5
	4∼5 million KRW	260	23.6
	5∼6 million KRW	131	11.9
	over 6 million KRW	70	6.3
Housing type	Apartment	514	46.7
	Townhouse	210	19.1
	Single house	370	33.6
	Other housing types	6	0.6
Geographical Districts	Capital area	520	47.3
	Southeastern area (Dongnam)	160	14.6
	Chungcheong	100	9.1
	Southern area (Honam)	160	14.6
	Daegyeong	120	10.9
	Gangwon	20	1.8
	Jeju	20	1.8
Residential area	Dong (urban)	800	72.7
	Eup/Myeon (rural)	300	27.3
Accessibility to foods	without LFA	1000	90.9
	with LFA	100	9.1

Note: 1 million KRW = US$833 ($1 = KRW1200, approximately) Source: Survey for this study (n = 1100).

**Table 3:** Average distance to the nearest grocery store by case (scenario) and upper-level administrative district (Metropolitan Si and Do in Korean).

	Case 1	Case 2	Case 3	Case 1-to-3 Ratio
Seoul	199	166	123	1.62
Busan	529	389	353	1.50
Daegu	758	653	560	1.35
Incheon	1909	1562	1348	1.42
Gwangju	752	722	618	1.22
Daejeon	595	554	523	1.14
Ulsan	1489	1229	1053	1.41
Sejong	1551	1411	1371	1.13
Gyeonggi-do	1285	1197	980	1.31
Gangwon-do	2251	2154	1968	1.14
Chungcheongbuk-do	1959	1867	1784	1.10
Chungcheongnam-do	1905	1593	1516	1.26
Jeollabuk-do	1800	1533	1480	1.22
Jeollanam-do	2084	1927	1872	1.11
Gyeongsangbuk-do	2555	2037	1958	1.30
Gyeongsangnam-do	2055	1680	1594	1.29
Jeju-do	1249	1200	849	1.47
Total	1804	1575	1460	1.24

Unit: meters.

**Table 4:** Number of the people with LFA in Korea.

Case	Number of People with LFA	Ratio	Number of Elderly People with LFA	Ratio
1	6,297,371	12.3	1,561,376	23.2
2	5,706,460	11.2	1,468,106	21.8
3	4,695,377	9.2	1,356,986	20.1

**Table 5:** Top 20 lower-level administrative districts with the lowest food access and their proportion of the people with LFA by Case.

Rank	Case 1		Case 2		Case 3	
	Lower-Level Administrative Districts	Ratio (%)	Lower-Level Administrative Districts	Ratio (%)	Lower-Level Administrative Districts	Ratio (%)
1	Ongjin-gun	83.88	Sinan-gun	80.27	Sinan-gun	80.10
2	Sinan-gun	81.31	Ongjin-gun	77.32	Gunwi-gun	73.61
3	Gunwi-gun	79.36	Gunwi-gun	73.87	Hampyeong-gun	71.35
4	Seongju-gun	74.73	Hampyeong-gun	71.78	Goesan-gun	68.66
5	Hampyeong-gun	73.35	Goesan-gun	70.04	Ongjin-gun	68.49
6	Sancheong-gun	71.16	Seongju-gun	68.84	Jinan-gun	68.02
7	Goesan-gun	70.92	Jinan-gun	68.67	Seongju-gun	67.39
8	Jinan-gun	70.27	Sancheong-gun	67.96	Imsil-gun	65.27
9	Uiryeong-gun	69.89	Imsil-gun	66.30	Sancheong-gun	65.16
10	Ganghwa-gun	68.68	Hadong-gun	66.19	Hadong-gun	64.60
11	Jindo-gun	68.29	Uiryeong-gun	66.18	Jangsu-gun	64.35
12	Hadong-gun	67.82	Jindo-gun	65.90	Jindo-gun	63.69
13	Imsil-gun	67.80	Ganghwa-gun	65.41	Ganghwa-gun	63.48
14	Cheongdo-gun	67.02	Haman-gun	65.00	Cheongdo-gun	63.43
15	Haman-gun	66.95	Cheongdo-gun	64.53	Uiryeong-gun	63.19
16	Bonghwa-gun	66.59	Jangsu-gun	64.35	Haman-gun	63.04
17	Jangsu-gun	66.09	Yeongyang-gun	63.47	Uiseong-gun	62.35
18	Uiseong-gun	65.67	Bonghwa-gun	63.07	Damyang-gun	61.78
19	Hapcheon-gun	64.84	Cheongyang-gun	62.91	Hapcheon-gun	61.66
20	Yeongyang-gun	64.80	Uiseong-gun	62.42	Cheongyang-gun	61.40
Total		12.31		11.16		9.18

**Table 6:** Average distance and one-way travel time to the grocery store mainly visited.

		Number of Respondents	Distance and One-Way Travel Time to Grocery Store Mainly Visited			
			Distance (Kilometers)		One-Way Travel Time (Minutes)	
			Mean	Standard Deviation	Mean	Standard Deviation
Top 5 lower-level districts with LFA		100	7.2	9.6	14.8	13.0
Lower-level districts other than the top 5 districts	Rural Area	100	3.5	3.9	11.4	8.2
	Mid-sized Urban Area	440	3.6	7.1	11.2	13.4
	Metropolitan Area	460	2.0	3.7	10.4	6.2
Total		1100	3.3	6.1	11.2	10.5

Note: *p*-value = 0.000 in Chi-square test Source: Survey for this study (n = 1100).

**Table 7:** Frequency of grocery shopping: administrative districts with and without LFA.

		Number of Respondents	Frequency of Grocery Shopping (%)						Total
			Once/Day	2–3 Times/Week	Once/Week	Once/Two Weeks	Once/Month	Less Than Once/Month	
Top 5 lower-level districts with LFA		100	1.0	23.0	46.0	19.0	7.0	4.0	100.0
Lower-level districts other than the top 5 districts	Rural Area	100	5.0	35.0	40.0	9.0	2.0	9.0	100.0
	Mid-sized Urban Area	440	1.1	26.1	53.0	16.8	2.7	0.2	100.0
	Metropolitan Area	460	3.0	39.1	49.6	7.0	0.7	0.7	100.0
Total		1100	2.3	32.1	49.7	12.2	2.2	1.5	100.0

Note: *p*-value = 0.000 in Chi-square test Source: Survey for this study (n = 1100).

**Table 8:** Frequency of grocery shopping via online: administrative districts with and without LFA.

		Number of Respondents	Frequency of Grocery Shopping Via Online (%)						
			Once/Day	2–3 Times/Day	Once/Week	Once/Two Weeks	Once/Month	Less Than Once/Month	Never
Top 5 lower-level districts with LFA		100	0.0	1.0	6.0	3.0	8.0	9.0	73.0
Lower-level districts other than the top 5 districts	Rural Area	100	1.0	4.0	2.0	1.0	2.0	12.0	78.0
	Mid-sized Urban Area	440	0.0	1.6	6.1	6.8	12.5	12.5	60.5
	Metropolitan Area	460	0.2	3.3	6.1	4.6	8.0	17.2	60.7
Total		1100	0.2	2.5	5.7	5.0	9.3	14.1	63.3

Note: *p*-value = 0.000 in Chi-square test Source: Survey for this study (n = 1100).

**Table 9:** Availability of a variety of foods required for nutritionally balanced dietary life in the grocery store that is mainly used.

		Number of Respondents	Fully Available (%)	Not Fully Available (%)
Top 5 lower-level districts with LFA		100	88.0	12.0
Lower-level districts other than the top 5 districts	Rural Area	100	97.0	3.0
	Mid-sized Urban Area	440	90.0	10.0
	Metropolitan Area	460	96.5	3.5
Total		1100	93.2	6.8

Note: *p*-value = 0.000 in Chi-square test Source: Survey for this study (n = 1100).

**Table 10:** Diseases related to dietary life and EQ-5D index.

		2017 KNHANES: National Average			CHS: Average of Top 10 Administrative Districts with LFA		
		All	Male	Female	All	Male	Female
High blood pressure	30 or older	26.9	32.3	21.3	34.3	30.2	38.2
Diabetes	30 or older	10.4	12.4	8.4	12.9	13.5	12.2
Obesity	19 or older	34.1	41.6	25.6	35.0	37.5	32.6
EQ-5D index	19 or older	0.963	0.970	0.958	0.923	0.947	0.899

Source: KNHANES [40] and Community Health Survey [39].

**Table 11:** Proportion of people reading nutrition fact label, quality of life index, prevalence of obesity by age group.

	Proportion of People Reading Nutrition Fact Labels		EQ-5D Index for Quality of Life		Prevalence of Obesity (Measured)	
	2017 KNHANES: National Average	CHS: Average of Top 10 Administrative Districts with LFA	2017 KNHANES: National Average	CHS: Average of Top 10 Administrative Districts with LFA	2017 KNHANES: National Average	CHS: Average of Top 10 Administrative Districts with LFA
All	33.2	14.3	0.963	0.923	34.1	35.0
19–29 years old	41.0	27.2	0.978	0.978	29.4	31.5
30–39 years old	41.6	32.0	0.979	0.982	33.4	38.0
40–49 years old	37.9	22.5	0.979	0.975	35.3	37.9
50–59 years old	26.3	14.1	0.962	0.962	38.0	37.4
60–69 years old	14.8	9.8	0.926	0.929	38.0	38.4
70 or older	5.9	2.8	0.868	0.830	34.7	29.9

Source: KNHANES [40] and Community Health Survey [39].
